# Corporate Weakness of Will

**DOI:** 10.1007/s10551-024-05804-x

**Published:** 2024-08-27

**Authors:** Kenneth Silver

**Affiliations:** Trinity Business School, Trinity College Dublin, 182 Pearse St, Dublin 2, Dublin, D02 F6N2 Ireland

**Keywords:** Corporate moral responsibility, Weakness of the will, Organizational control

## Abstract

Proponents of corporate moral responsibility take certain corporations to be capable of being responsible in ways that do not reduce to the responsibility of their members. If correct, one follow-up question concerns what leads corporations to fail to meet their obligations. We often fail morally when we know what we should do and yet fail to do it, perhaps out of incontinence, akrasia, or weakness of will. However, this kind of failure is much less discussed in the corporate case. And, where it is discussed, the view is that corporations are less prone to weakness. Here, I argue that proponents of corporate responsibility should say that corporations can and often do instantiate weakness of the will, and that this is important to recognize. Weakness of the will requires certain capacities that these proponents typically take corporations to have. And once this is appreciated, we can assess how corporate weakness might proceed differently than how it does for individuals. We can also begin a conversation about how best to meet the distinctive challenges for recognizing and correcting corporate weakness, using a number of resources from management scholarship.

“…the incontinent person is like a city that votes for all the right decrees and has excellent laws, but does not apply them…” (Aristotle, *Nicomachean *Ethics, Book VII, Ch. 10, §3: 20, 21)

## Introduction

Can corporations, as potentially responsible agents in society, instantiate anything like *weakness of will*? And what would be the significance of this if so? Management scholarship has considered the question of the causes of corporate misconduct (Greve et al., 2010), as well as mechanisms for the attribution of corporate irresponsibility (Lang & Washburn, 2012). So, it may seem perfectly plausible for firms to act wrongly in this way, and for us to attribute the wrong conduct to them. This picture might be appealing to those working within the behavioral theory of the firm in particular, which takes seriously the idea that how firms decide and act can be modeled on how individual agents might behave (Cyert & March, 1963; Gavetti et al., 2012). However, whereas the behavioral theory is often read analogically—providing a way for us to theorize about the firm by analogy to deliver insights (Ketokivi et al., 2017)—our question here is meant *literally*. Is it possible for firms to act in ways that don’t just look like cases of weakness of will if firms were agents like us, but to literally instantiate weakness of the will, perhaps even if their employees do not, and for it to be appropriate to hold firms morally responsible for this?

Within normative business ethics, there is a long-running debate over whether it is ever appropriate to blame corporations *themselves*, or to take them to be responsible in any way that does not somehow reduce to the responsibility of their members. Opponents of corporate moral responsibility (‘CMR’) are likely to say that whatever blame we feel is really meant to be targeted at employees (though we may not know which ones) (e.g., Hasnas, 2010; Velasquez, 1983). These are appropriate responses if we think that the firm itself is just the collection of individuals, or that the firm itself cannot act in ways for which it can be responsible, or if we think that the firm cannot be the appropriate target of blame (Sepinwall, 2017; Shoemaker, 2019).

Alternatively, proponents of CMR hope to vindicate many instances of corporate responsibility. According to proponents such as Peter French, Tom Donaldson, David Silver, Philip Pettit, Christian List, Carol Rovane, David Copp, Kendy Hess, Stephanie Collins, and many others, at least some corporations are capable of acting and being morally responsible for what they do (and not merely legally culpable). That is, firms are literally responsible, and it is possible for a firm to be responsible for some misdeed even if none of the individuals responsible for them are similarly responsible (Copp, 2006). It is of course up for debate exactly *which* corporations could qualify as true corporate agents. Still, the thought is that some firms (likely complex, structured organizations) will qualify as responsible for what they do over-and-above the responsibility borne by individual members of them.

Given this, only proponents of CMR will potentially be in a position to affirm corporate weakness of will, or what I will call ‘corporate weakness.’ (Opponents will attribute any responsibility for apparent weakness to certain employees.) However, it is not obvious that such proponents will be willing to recognize corporate weakness. Few proponents actually consider the sources of corporate immorality; instead, their focus is entirely on whether corporate moral responsibility can be affirmed *at all*. And what little discussion there is of this topic suggests a rejection of corporate weakness.

Peter French (1979) was an early and staunch advocate for corporate moral responsibility, but, for reasons we will discuss, he maintains that organizations like corporations are much *less* likely to act weakly (French, 1995). Since then, French’s point has passed without much discussion (Tollefsen, [2008: 9] remarks on this feature of French’s view, but leaves it unchallenged.).^1^ One notable exception is Pettit (2003)’s positive argument for group/corporate akrasia, also to be discussed below. However, Pettit doesn’t recognize French’s concerns, and there is a more general failure to appreciate or handle what it is about corporate weakness that might strike us as especially odd.

Stepping back, it is not surprising that corporations would be thought to be less prone to weakness. Acting weakly often has a certain feeling about it. We are giving in to *temptation*. It feels good, but leaves us with feelings of guilt and regret. At the very least, it is internal and tumultuous. Corporations, however, act through the execution of established rules and procedures. They act, but likely without the burden of emotions or phenomenal experiences. So, perhaps the natural view of the proponent of CMR is that *corporations cannot act weakly*. The problem with this is that it is not hard to imagine many cases where weakness is most natural diagnosis.

Consider a corporation (Chairs 4 All) with an explicit diversity & inclusion mission statement^2^:

Chairs 4 All is deeply committed to championing a plurality of lived experiences in the workplace, empowering our people to express disparate opinions and perspectives and providing tools for professional growth and meaningful contributions. Diversity in hiring and promoting is among the highest priorities of Chairs 4 All, as we recognize the value of diversity for developing a sustained competitive advantage in the global marketplace.

Suppose that an independent audit was done of Chairs 4 All in 2023 and found that the company was *less* diverse in its hiring and promotions than 90% of firms either of its size or in its sector. Moreover, since this mission statement was adopted 7 years ago, say, the company has not improved by any measure of diversity or inclusion. Now, what should we think about this complete failure of Chairs 4 All to act in a way aligned with their mission statement?

Some of this, much of it even, may come down to individual failures at the level of hiring managers, interviewers, or line managers. These individuals may be led in their hiring and promoting practices by their own prejudices or biases. They may be led to hire in ways that are not diverse by the same biases in resume reading, for example, with which we are now quite familiar (e.g., Derous & Ryan, 2017).

To the extent that the employees are acting in this way, they will have acted wrongly. They are obligated not to be biased in these ways. And we may also think that there is an obligation to their employer that employees are failing to fulfill in not aligning their conduct, as agents of the firm, with the mission statement. Apart from this bad conduct of employees, though, it may be natural to think that the firm itself bears some responsibility for failing to comply with its own standards, its own expressed values. In fact, this is surely something that proponents of CMR will want to affirm. Thus, the natural diagnosis is that Chairs 4 All has acted weakly in some way. The moral failure of the firm at issue in the case does not seem to be a matter of the firm’s making the wrong judgment about how to act; instead, it seems to concern their inability to adequately enforce the commitments they have made.

Proponents of CMR are left, then, as either having to find some other way to characterize the case of Chairs 4 All or else as being unable to recognize it as a corporate wronging, despite it clearly being a case for them to capture. I will argue that this issue should be resolved by allowing for corporate weakness. Corporations as agents can be weak-willed or akratic. As a matter of fact, we will see that firms may be even *more* prone to akratic actions than we are as individuals.

I will start by considering different views about what weakness of the will consists in, and so how much is required to instantiated it. Then, in the following section, I will argue that proponents of CMR likely already accept corporations as having the states required to instantiate weakness. We will see that weakness need not presuppose phenomenology even in our own case, and so it may be possible for firms to act weakly without it. Moreover, I will draw out and answer the separate issue mentioned above concerning how rule-governed firms seem to be. Plainly, being rule-governed generally is compatible with instances of failing to be rule-following.

If firms instantiate weakness, there might be differences between how firms and individuals act weakly. Moreover, there may be special challenges in the recognition and correction of corporate weakness. The third section explores these potential differences with corporate weakness, and the final substantive section considers these additional challenges. This discussion leads to several insights. Corporate weakness is most likely to manifest as a matter of the firm’s habitual agency, or how the firm and its agents act habitually. It is especially challenging to distinguish corporate weakness from disingenuously made corporate resolutions, or from things done by rogue employees. But these challenges have interesting analogues in the case of our weakness, and they can be addressed by being sufficiently connecting to management literatures focused on organizational control and authenticity. There is a further question about how to control employees well without *dominating* them, and I suggest that this can be achieved by more intentionally adopting and communicating the firm’s strategy and value orientation.

## What Does Weakness of the Will Involve?

It will be hard to assess whether corporations can suffer from weakness if we do not have some conception of what is involved in acting weakly on hand. Unfortunately, part of what is at issue in the literature is precisely what is involved internally in agents acting from weakness or akrasia. (Or, for that matter, whether weak-willed action and akratic action are distinct phenomena. See Holton 1999, Mele (2010), and May & Holton (2012).) Authors tend to proceed by considering cases of paradigmatic weak-willed action and introspecting the agent’s psychology.^3^ So, let’s begin by considering the following case:

**Chocolate Chip:**

Chip loves the taste of chocolate. Still, he feels that he’s been indulging a little too much lately, so he decides not to have any chocolate treats at a party on Saturday where he knows there is likely to be chocolate. Sure enough, there is chocolate at the party. And, sure enough, Chip sees the chocolate and indulges. Later, he regrets his behavior. The pleasure was quick to fade, but his knowledge of his own weakness remains.

This vignette depicts paradigmatically weak-willed behavior, but it is told in such a way that we can imagine that there are different elements to what is happening within Chip psychologically. Within the literature, we can in fact find at least five different stories for what might be going on in the case:

1. Perceptual illusion
2. Corruption of the Will
3. Overwhelming desire
4. Lack of or insufficient desire
5. Reconsidering a resolution
6. Acting out of habit

In the *Protagoras* (352-356c), Socrates argues that weakness of the will is impossible. Weakness of the will involves one’s knowingly acting contrary to one’s better judgment, but Socrates maintains that one cannot act way in full view of another act that one genuinely believes one ought to do instead. Persons are not knowingly evil. To account for *apparent* cases of weakness, then, Socrates maintained that they were in a kind of perceptual or ‘evaluative illusion’ (Watson, 1977: 319), perhaps involving how comparatively vivid the hedonic qualities of proximate pleasures relative to distant ones (Obdrzalek, 2023). The agent is not acting against what he takes to be best; the agent is tricked by the tempting visual quality of the chocolate into reevaluating what he takes to be best, and then acts accordingly. On this story, although Chip has decided that it is best not to eat chocolate at the party, we can suppose that he gets to the party, looks at some chocolate, and thinks, “Now wait a minute. I don’t remember chocolate looking *that* good. I think I might have undervalued the significance of the joy that I would get from eating this chocolate. And, correcting that error, it seems clear that what I ought to do is to eat the chocolate.”

There does seem to be something deeply true about the Socratic story. Agents often display a bias for near-term benefits over greater long-term rewards (which is much discussed in economics and philosophy). Arguably, what is happening is that it is easier for us to attend to the value of what’s in front of us, and so we are likely to give it more weight in our decision-making. Still, there are other stories for what might be going on in the case that do not end up denying the reality of weakness of will.

Considering (2), it may be natural to think that all agents possess a Will, and temptations threaten to corrupt it. Understanding what this looks like psychologically or metaphysically can be quite challenging, with few scholars these recent decades being willing to reify the Will as something existing and corruptible. If we were to go in for it, though, it would seem reasonable to maintain that the Will, and exertions thereof, involve something phenomenological, with there being a feeling of what it is like to will an action to completion, or fail to. More often, though, discussion of the Will is connected to *willpower*, where this is understood in ways apart from the Will itself. Instead, it is taken to reduce to other psychological features. It may be a matter of psychological effort that stops one from reconsidering a resolution (Holton, 2003), or else involve psychological processes of suppression or resolve (Ainslie, 2021), or else be wholly (and problematically) metaphorical (Koi, 2024).

In contrast, (3) is likely the dominant conception of what normally happens in cases of weakness of will. On this picture from Donald Davidson (*op. cit.*), Chip had judged it best not to eat chocolate and yet eats it, acting contrary to his better judgment, or his all-things-considered judgment. And this is likely due to a strong desire for chocolate. It was the strength of the desire that somehow tips Chip toward concupiscence. There is a temptation here to say that in these cases the agent is *overwhelmed* by their desire. Put this way, it again has a phenomenological flavor. Chip is reacting to an extremely strong *feeling*. However, the model need not be understood in this way. If the agent was truly overwhelmed, then it starts to make us wonder whether this really is a sincere action for which the agent is responsible, as opposed to an instance of compulsion. (We will return to this issue below.) And, relatedly, it is often observed that agents can remain quite calm as they act weakly.

Instead of modeling the agent as overwhelmed by a desire for what they judge to be the wrong thing, moving on to (4), we might instead imagine that the agent simply insufficiently desires or is motivated to do what they judge to be the right thing. In the *Nicomachean Ethics* (1999, Book 7, ch. 1–10), Aristotle draws out this picture in a lengthy discussion of incontinence and weakness. He distinguishes it from bestiality, intemperance, softness; he considers whether the incontinent person knows what he is doing when acting; he even explicitly contrasts his view with the Socratic picture. About weakness in particular, he says, “For the weak person deliberates, but then his feeling makes him abandon the result of his deliberation…” (*ibid.*: ch.7, §8: 21–22).

On an Aristotelian model of moral motivation, virtuous agents desire and are motivated to act in accordance with reason. Self-controlled agents may additionally desire to do the wrong thing, such as pursue un-choiceworthy pleasures, but they will nevertheless be motivated by the right desire to act rightly. Genuinely akratic agents, by contrast, are inadequate with respect to having the right kind of motivation, the desire to do the right thing. There are some interpretative challenges concerning whether Aristotle took akratic agents to *entirely lack* this positive desire (as Henry [2002] argues) or whether akratic agents do potentially have the right desire, but where that desire is insufficiently strong and so outweighed by a desire to do otherwise (as Stoyles [2007] suggests). Either way, though, we are left with a model of akasia in terms of the having and lacking of the right mental attitudes.

Whereas the Davidsonian and Aristotelian stories involves this simple interplay between the desires of the agent, more complicated stories have been offered more recently in terms of the concept of intention and resolution. For Richard Holton (1999, 2003, 2009), what is significant for a case of weakness of will is that the agent has formed some resolution about what they are to do, but then they are inappropriately brought to reconsider their resolution. (This, then, is (5)’s interpretation of what is going on in the case.) Since Davidson, the literature has come to recognize the many mechanisms that agents fashion as a means of achieving diachronic control. In particular, resolutions are a form of self-binding that we use so that we are able to settle our minds on what to do. This frees us up cognitively, allowing us to avoid endlessly, anxiously weighing our options right until the last moment. So, on this picture, Chip has formed a resolution not to eat chocolate at the party, and what’s critical is that something has happened to lead him to reconsider whether to eat the chocolate.

As a final alternative (6), perhaps Chip just has this habit of eating chocolate in front of him if he’s not careful, and he failed to attend to his intention to not eat chocolate as he habitually consumed it. Whereas most of the literature focuses on these perhaps intense experiences of giving into temptation, Silver (2019b) suggests that there’s a kind of weakness possible when acting out of habit, where one fails to be sufficiently vigilant about what we have intended to do. Failures of vigilance and self-control can be moral failings (Murray, 2017; Murray & Vargas, 2020). But we are creatures of habit—some bad and some good. We can try to change our habits or intend to act a certain way despite them, but if we are not careful, then we may act contrary to our intentions or judgments because of them.

For our purposes, we do not need to come to a view of what the right psychological picture is in the case of Chocolate Chip. Each of these psychological stories can be made to sound somewhat plausible in the case. The literature on weakness of the will primarily concerns which of these psychological stories is essential for truly weak-willed action. However, we do not need to come to a full view of what constitutes weakness of will for our purposes.

Addressing this fully involves coming to terms with a number of interesting issues. Among others, we would need to understand the significance of different kinds of judgments, have a view of how desires relate to intentions/resolutions, determine the psychological reality and significance of willpower, discover the degree to which judging a choice correct immediately motivates us to enact it, and so on. But even without resolving these issues, most philosophers will accept that most of these psychological stories could have been what happened in Chip’s case and certainly do happen regularly in life. More important is recognizing the kinds of views out there, and the psychological/cognitive machinery that they tend to require.

There were any number of ways psychologically that Chip could have failed to act in line with his decision not to eat chocolate. In each of them, though Chip knew what he had to do, he failed to be properly motivated to do it. Our question is, assuming that certain corporations can act and be obligated to act in certain ways, are there any similar ways that they can be led to fail to meet their obligations?

## Do Corporations Have What It Takes?

I think the clearest path toward answering whether corporations can act weakly involves considering whether explanations for certain corporate conduct can be given that is similar or analogous to Chip’s actions. However, before pursuing this, I first want to acknowledge and consider the limitations of the earlier instance of direct advocacy for group/corporate akrasia alluded to in the introduction. Pettit (2003) distinguishes between different kinds of groups, eventually focusing in on ‘self-unifying cooperatives’ or group agents like certain organizations and corporations (78). He argues that groups like these can act akratically and perhaps often will based on how they form decisions.

Groups, like individuals, can be rationally constrained on the basis of their previous decisions. Pettit shows how groups can have procedures for arriving at decisions (such as majority voting) that can lead to cases where group rationality requires deciding and acting a certain way, but many or even a majority of the individuals in the group may disagree, thinking the group should act differently. And so, these individuals may be motivated to act against what it is rational for the group to do. Importantly, this need not be a matter of these individuals themselves acting akratically.^4^ They may act as they see it in the best interest of the world, albeit threatening group irrationality. As Pettit says, “Personal virtue is as likely as personal vice to source recalcitrance towards a collectivity. Virtue in the individual members of a group may make for akrasia in the group as a whole” (84).

This is an important kind of case to recognize. If it is group akrasia, then Pettit thinks this shows that akrasia need not be a matter of a failure of executive control (89–93). And Pettit’s focus on it is understandable insofar as it relies on certain challenges with collective decision-making (i.e., the discursive dilemma) that he has long discussed. However, there are a few problems.

First, the kind of case Pettit considers is ultimately quite narrow. He imagines a group of individuals who each contribute to group decisions, and akrasia for him basically involves defectors, or when individuals act in full view of what they know the group should do (rationally speaking) and for their own reasons. If this counts as group weakness, I don’t think it is likely to be the usual case in corporations. We will see more about why below, but essentially the point is that corporate weakness may be instantiated by individuals in the firm with no standard input into corporate decision-making, who continue to act for corporate reasons, and who are insufficiently aware of what the firm is rationally required to do.^5^

With that said, I am also not confident that the group conduct as Pettit describes it genuinely is akratic. It may be, and I will suggest how below, but one reaction is that it is not genuinely weak-willed group *action* if individuals are going rouge and acting on their own motivations. Perhaps their behavior is attributable to the firm insofar as they act in their role as employees, and perhaps firms must be ready to take responsibility for this conduct, but that doesn’t mean that it is best understood as akratic or weak-willed behavior.

Pettit has a surprisingly low bar for akratic behavior; it simply involves failing to act in a required manner that is in some way recognized (68). The problem is that *compulsive* behavior might fit this characterization, and many scholars will argue for a difference between compulsive and weak-willed conduct. We will return to this issue below, but for now it should leave us suspicious of Pettit’s conception of group akrasia. With individuals acting on their own motives against what is it rational for the group to do, in what sense is the group really suffering from a kind of weakness (perhaps inappropriately privileging some reasons over others), as opposed to simply failing to be responsive to its own reasons?

I think the best way to proceed is by sticking closely to exactly how scholars have characterized weak-willed behavior, as we saw in the last section. We can be reasonably sure that corporations act weakly if they perform actions which have explanations that are analogous to any of the explanations of Chip’s action. Unfortunately, there are two kinds of challenges to thinking that they ever do perform actions with the same kinds of explanations.

First, the explanations of Chip’s misconduct were pointedly psychological. They involved a suite of mental states that we may think corporations or other kinds of groups simply cannot instantiate. For starters, a number of the explanations required states such as judgments (that one course of action in superior), desires, intentions, and resolutions. Apart from these basic action-involving mental states, however, we might also think that a host of even more sophisticated mental faculties are required. If we think that all weak-willed actions are a product of misapprehension or some kind of illusion, then this seems to require certain perceptual faculties. Authors such as Richard Holton also take there to be such a thing as willpower that plays an important role in agent’s self-control. And others take certain emotions to play an important role in weak-willed action (Tappolet, 2003), such as the guilt of acquiescing to temptation. In short, the more cognitively involving we take instances of weakness to be, the less likely we are to think corporations are capable of instantiating it.

Second, we might think analogous cases of even possible group weakness will be hard to find, because group agents tend to be constructed in ways to avoid weakness. This was precisely the basis on which Peter French thought cases of corporate weakness would be unlikely. He says, “The [Corporate Internal Decision] structures of corporate actors can be designed so that they are not prone to weakness of will. They can be specifically built to override human weakness of will” (*op. cit.*:47). The thought is that corporate behavior is largely governed by procedures and rules of the corporation. Given this apparent blind, rule-following nature, there will not be cause for corporations to be bumped off of course and into akratic action. The group can control itself in ways and to a degree that individuals cannot.

Let’s consider these challenges in turn.

### Can Corporations have the Right Mental States?

This first objection feels most pressing, but in fact I think it will fall flat for proponents of corporate responsibility. Though the explanations of Chip’s misconduct were psychological, it was unclear how much or what kind of psychological states are *really* required for weak action. And even if a hefty amount of psychological baggage is required for Chip and other individuals like him to act weakly, perhaps the same would not be required of corporations.

In my own case, I often feel the phenomenal force of temptation. I feel pulled toward the object of my desire, steeped in the knowledge of the pleasure that accompanies its satisfaction. Put this way, it can be hard to see that corporations would be capable of being weak willed. After all, most proponents of CMR deny that corporations are genuinely phenomenally conscious (e.g., Hess, 2013; Baddorf, 2017; *c.f.*, Schwitzgebel, 2015; Silver, 2019a:261–3). So, if it is only conscious experiences that lead to weak-willed behavior, then firms would not act weakly. However, it might be inappropriate to generalize from our own experiences. That our own weak-willed behavior feels a certain way may be a typical feature of it for us, but maybe corporations do not *need* this kind of baggage to act weakly.

Thinking back to the stories we saw trying to explain Chip’s weak-willed behavior, note that most of them involved certain mental states within Chip, but not necessarily the phenomenal quality of those states. What matters was that Chip had made a resolution and then was drawn to give it up, or that Chip was led to reconsider a judgment. (Not that there was something that it was like for Chip to experience coming to a resolution or abandoning it.) The reason for these changes for Chip may be a matter of feeling the pull of temptations. But apart from that pull, the same effect may occur merely given a reflection upon Chip’s incentives, perhaps along with certain biases Chip might have, such as a tendency to overweight the satisfaction of satisfying short-term desires as opposed to long-term ones. These biases and changes in judgment can be recognized apart from any phenomenology associated with them, so there is reason to think that the states that may be necessary for instantiating weak-willed behavior may not require phenomenology.

Further, there is good reason to think that proponents of CMR will take firms to have the necessary states. Any such proponent is likely to say that groups are capable of coming to judgments regarding what to do. In fact, that corporations and group agents can arguably come to collective judgments about what should be done that are not necessarily shared with any of the individual people in charge of them is one of the primary motivations in the literature for accepting corporate agency (List & Pettit, 2011; Pettit, 2007, 2011). So, if firms can have these judgments but fail to act on them, then that may be all that is necessary.

(If weakness is understood on the Aristotelian model of being insufficiently motivated by the judgment – having judgments and *not having* the right corresponding attitude that leads to action – then this will be easy for firms to instantiate. In fact, this is likely the best interpretation of group akrasia as Pettit (2003) discusses it. There is a constraining group decision, but there is no corresponding group attitude to motivate the individuals in that direction apart from their own motivations.)

In each case of some apparently necessary psychological component, we will likely be able to either make the case that groups do instantiate some version of that state or else argue that those states are not required for weakness.^6^ As an example of the latter, we could argue that while certain emotions may naturally accompany our own descent into weakness, perhaps groups may act weakly and issue an apology thereafter without having to feel these emotions. (Doucet [2016] argues that emotions like regret need not even accompany our own instances of weakness.) As an example of the former, while we might accept for the sake of argument that groups lack some phenomenal kind akin to our willpower, we might point to other forms of self-binding and self-control that can stop weak-willed action (or fail to). Collins (2023: ch.4) expressly argues that organizations can be blameworthy on accounts that require acting from a certain volition or will, and she draws out how Held (1970), List and Pettit (2011), Tollefsen (2015), and others similarly suggest firms and group agents are blameworthy assuming similar volitional requirements for blameworthiness.

If we wanted to be extremely bare bones about it, we could even argue that all that is really required for firms to act weakly is for them to have most reason to behave a certain way, be in some way in a position to act in light of that reason, and yet fail to do so. All that would be required for weak-willed action on this conception is an ability to be responsive to reasons even if the firm itself lacked mental states (Silver, 2022). However, since most proponents of CMR are already committed to some degree of corporate mentality, there is little reason to think why such proponents would not take firms to have the mental states necessary for weak-willed behavior.

Clarke (1994), Tollefsen (2002), Copp (2006), and others argue (on the basis of their acceptance of functionalism in the philosophy of mind) that groups can instantiate mental states such as beliefs, desires, and intentions.^7^ Tollefsen (2008), Collins (2018), and Silver (2019a) consider how corporations can be construed as having emotions, and Björnsson and Hess (2017) even argue that groups can instantiate reactive attitudes like guilt. If these proponents were to agree that groups cannot act weakly, it would likely not be on the basis of their lacking the requisite mental states.

None of this will satisfy opponents of CMR, who regularly deny claims that corporations can themselves have mental states of any kind. They do not *just* lack phenomenal consciousness; it is also pure metaphor to suggest that firms can ‘want’ things or ‘intend’ or ‘judge’ this or that. Without these mental states, surely firms will not act weakly. This is not the place to adjudicate this larger question. The point is that those who *already* allow for sufficient corporate mentality for firms to behave in responsible ways have likely already admitted the components necessary for firms to act weakly. So, proponents of CMR should accept the possibility of corporate weakness of will.

### Are Corporations Too Rule-Governed to Act Weakly?

Still, even if firms have all that it takes to act weakly in principle, should we really think that they often do? Perhaps corporate agents are organized to ensure that acts judged to be best and intentions set will be carried out by the corporates in virtue of the procedures that the firm has put in place for carrying out its actions. So, perhaps even if firms could act weakly, they typically do not.

I see why authors have been drawn to this opinion. Rules and procedures are so integral to corporate life that we may take them to be even partially constitutive of the corporation. For someone like Peter French, corporate conduct is entirely a matter of employees acting within their roles as defined by these corporate structures, so it must seem impossible to countenance corporations legitimately acting yet in ways not in conformity with the procedures that have been laid down. Nevertheless, I think even a cursory consideration of real corporate life reveals just how prevalent cases exactly like this can be.

If we consider some of the most morally egregious corporate offenses, we are just as likely to find a breakdown in the procedures meant to secure adequate self-control that we see in the case of individuals. Consider the following case,

**Volkswagen:**

Suppose Volkswagen is a car manufacturer that wants to produce cars cheaply and efficiently and doesn’t care about the environmental impact of those cars. However, Volkswagen knows that it is against the law to sell cars that fail to meet certain fuel efficiency standards, and so Volkswagen sets a policy of delivering high-quality cars that meet these standards, even if it would be easier to cheat the fuel efficiency tests than it would be to cheaply meet the standards. Sure enough, it is easier to cheat the tests than cheaply meet the standards. And, sure enough, Volkswagen cheats on the fuel efficiency tests. After being caught, Volkswagen faces hefty fines, a loss in public trust, and issues public apologies.

The example is not subtle, but it should make the point loud and clear. If groups like corporations are capable of acting wrongly and being responsible, then the case of Volkswagen’s fraud surely is a paradigm instance of wrong action. And it is not reasonable to think that Volkswagen acted wrongly because it did not adequately appreciate the moral reasons that it had to comply with the law. Instead, what seems to have gone wrong is an inability to translate the corporate judgment of legal compliance into action.

Just as in Chip’s case, there are a number of ways that the story might go for how this failure occurred. Perhaps Volkswagen judged that it ought to comply with the law, but this judgment did not translate into motivation for the corporate agents to act it out (the Aristotelean model). Or perhaps Volkswagen’s background desire for profit motivated corporate agents to commit fraud. Or perhaps Volkswagen formed a resolution to remain within the law that was later collectively abandoned or changed (even if not formally). Or, finally, perhaps Volkswagen had practices of cutting costs and tampering with efficiency test equipment that were so ingrained that they were not appropriately sensitive to a resolution to comply with the law. (I won’t take a stand on the right story in this particular case, but we will see in the next section how this last story is the kind of story I think most commonly leads to corporate weakness.)

In this case, we don’t need to know which kind of mechanism actually led Volkswagen to fraud. Patsioti-Tsacpounidis (2023: 429) compellingly argues that this is an instance in which corporate wrongdoing stems from the greed and akrasia of managers. For our purposes here, that may well be right. But all we need to know is that Volkswagen did the wrong thing and because it was unable to have its practical judgments guide its behavior. Despite surely having many rules and procedures for how its employees should conduct itself, at the end of the day these rules were no better at tempering Volkswagen’s immoral conduct than the principles that individuals purport to follow. More generally, we can appreciate that the mere fact that groups are often rule-governed in principle does not mean that they will *remain* as rule-governed in practice.

We saw above how French maintained that corporate weakness was unlikely because they can be designed to avoid weakness. Well, have they been? As we see above, compliance departments based entirely on the enforcement of *legal* rules on the firm can be unsuccessful. I am optimistic with French that perhaps we could use the malleability of the corporate structure and our knowledge of moral psychology to *engineer* firms this way. But we surely have only barely begun this project. And this paper is a clear step: We need to know *that* corporations can act weakly, then we need to investigate why and how corporations most often act weakly. So, we should not think that firms *as they are today* are any less prone to weakness just because their structure is subject to revision.^8^

## What is Distinctive About Corporate Weakness?

Even accepting that corporations can act weakly, this is not to say that corporate weakness of will is *just like* weakness of will as it occurs for individuals. There will be differences, and attending to these differences could be significant when it comes to our ability to recognize and potentially guard against corporate weakness.

Most notably, it may well be that the path to corporate weakness as it most often occurs, or how corporations act weakly, may diverge from the typical process of individual weakness. We saw a number of stories given above for what is happening in the case of individual weakness of will. There is now a large debate about *which* of these stories picks out what is truly akratic or true weakness of will, or whether these come apart. Nevertheless, I take each of these processes to be possible for individuals, and for them each to have problematic results, even if they are not all properly called ‘weakness of will.’ Even if they are each possible, though, we might wonder whether certain of the routes are more common than others.

Notice how often individuals seem to rationalize their choices as they make them. As I abandon my resolution to run every day, as I am tired from the first day of running, I tell myself that running every day is probably a bad idea anyway—surely it’s not good for the knees; surely there are more efficient exercises. Similarly, when Chip eats the chocolate, though he may ultimately regret his behavior, at the time he may well change his mind and decide that a little chocolate is not a bad thing, and what’s life for anyway? Generally, individuals seem liable to switching their judgment in the moment of temptation and even claiming that it was actually the rational thing to do. We talk ourselves out of things.

In contrast, I am not sure how this kind of process might proceed in a corporation. When confronted with the results of the audit, I do not think that the executives of Chairs 4 All are likely to say that the firm had acted well and simply failed to update its mission statement. This is not to say that this kind of process *can’t* happen in corporations. But I think it is much less likely to happen in firms than in incontinent individuals, and for good reason. Even if we think corporations literally have intentions to act, and form judgments, and can instantiate states similar to guilt or shame, even this is a far cry from think that corporation instantiates first-personal, dialogical states of mind of the form, “Yes, why shouldn’t I?”.

As individuals, we have a running train of thought and a psychological need to appear rational to ourselves as agents. That is, we represent ourselves *to ourselves* in thought. These indexical or *de se* thoughts occur to us consciously, and they play a role in our thinking and acting. Now, it is controversial exactly what is distinctive about *de se* thoughts or what role is played by them (Torre, 2016). But *de se* thoughts of this kind do seem important for this way of instantiating weak-willed behavior.

In general, I am sympathetic to those arguing that corporations token many different kinds of mental states, but I think it is unlikely that they often instantiate indexical thoughts of this kind. Even if corporate spokespeople can make statements that refer to the firm as a whole in the first-person plural (e.g., “We here at Chairs 4 All promise to…”), and even if an appropriateness condition on statements of this kind involved their reflecting some kind of attitude with *de se* content, this is far short of ongoing *de se* thoughts occurrently structuring how corporations engage in reasoning and decision-making. That is to say, even if they *can* have these thoughts, it has yet to be argued for explicitly, and it is not clear that these thoughts would play the same role in their weak-willed behavior has our *de se* thoughts play in our behavior.

In contrast, firms seem much more likely to act weakly via something like habitual action.^9^ There is now a substantial literature within management scholarship on routines within organizations (e.g., Becker, 2004; Pentland & Rueter, 1994), and routines are often connected in some way with habitual action within firms (usually the habitual action of employees) (Makowski, 2021). While more would need to be said, a proponent of CMR should generally be open to the possibility of corporate habitual agency, and likely ready to identify corporate habits with some organizational routines.^10^

All too often, executives set the plan for the organization, but other members proceed with their own momentum. The larger and more complex the organization, the more inevitable this becomes. Employees constitute corporate conduct, and so the firm is inclined to act as it always does. Without special care, corporate conduct generally may be somewhat insensitive to what the firm technically judges to be best, perhaps what has only been affirmed in the C-suite. Our original example seems to be of this kind. Executives have established a mission statement, but they seem to have failed to sufficiently bring the whole firm to act in line with it. This is not to say, in line with Pettit’s model, that employees are going rouge—acting on their own motives for what they judge the firm should do regardless of corporate rationality. It’s just that not enough have been done to thoughtfully change hiring practices, for instance.

When employees enact corporate routines, which new corporate decisions and missions have failed to unsettle, these individuals are in some sense still be acting for the corporate reasons the routine was originally established, not their own rouge motivations. Of course, employees can go rouge, and then there are interesting questions about whether to attribute their agency to the firm and how best to hold the firm liable. These are ongoing issues especially within social ontology and the philosophy of corporate criminal law. But that’s not the best interpretation of what is going on in the case of Chairs 4 All, or indeed in many cases where firms fail to move themselves to act in line with their own judgments.

Large organizations require a substantial regulative infrastructure to avoid the most basic instances of akrasia. Such infrastructure is expensive and can only do so much. (We will return to the question of how much it should do below.) It is hard enough to minimally ensure *legal* compliance, let alone moral compliance, or complete compliance with quarterly shifts in managerial directives. When the left hand doesn’t know what the right hand is doing, *but should*, then this too is a kind of weakness. And this kind of coordination is especially challenging for international organizations operating across many different local contexts and cultures. Such an organization may commit itself to a certain course of action but lack the self-control to implement strategy across its businesses.^11^

While individuals may exhibit this kind of habitual weakness (I certainly do), firms seem more prone to acting weakly in this way. Still, assessing the problem is the first step toward solving it. If we can judge not only that corporations can and do act weakly, but that the source of their weakness often stems from this misalignment between the judgments or pronouncements of executives and conduct resonating out through the organization, then we can see a *prima facie* path toward steeling corporations against weakness.

What is perhaps especially promising with this kind of approach is that it suggests a natural integration of work within management scholarship into business ethics. In the past few decades, there is a whole research stream focused on *organizational control* (Cardinal et al., 2017; Flamholtz, 1996; Flamholtz et al., 1985; Ouchi, 1979; Ouchi & Maguire, 1975; Sitkin et al., 2010). Scholars have focused, for instance, on how to think about control through organizational structure (Ouchi, 1977), or through algorithms (Kellogg et al., 2020), or how different controls lead to different performance outcomes (Sihag & Rijsdijk, 2019), or how control is influenced by the way in which managers construe problems the organization is trying to solve (Zhong et al., 2022). So, whereas business ethics can articulate what is bad corporate conduct and perhaps what constitutes corporate weakness, it may be that management scholarship has already furnished the tools for better articulating the nature of the failure in question and what can be done to fix it.

## Outstanding Challenges to Recognizing and Correcting Corporate Weakness

The task of this paper has been to make the case for the claim that proponents of CMR should accept the possibility of corporate wrongdoing as a matter of weakness, as well as against the thought that surely firms are much less prone to weakness than we are. I think this task has been achieved. Practically, though, the more that can be said about recognizing and correcting corporate weakness, the better. I suggested just above that a practical path forward would involve dutifully pointing to cases of weakness, and then using work on organizational control to help correct this weakness or avoid it in the first place. However, there are a number of challenges with engaging in this process. In this section, I want to consider these challenges. None of them undermine the central point defended about the possibility (and commonality) of corporate weakness. Still, acknowledging and engaging with them is an important part of determining how best to take action against corporate immorality.

### Challenges with Recognizing Corporate Weakness

It is not easy, even in the individual case, to recognize some conduct as an instance of weakness of will. And issues are magnified in the corporate case. Let us consider three ways in which it is hard to distinguish corporate weakness from some other phenomena.

First, weakness of will is an agential failing for which a firm might be blameworthy, but merely acting *irrationally* might be seen at times as more accidental than actually bad. It might not be good, and it could be worth recognizing and avoiding, but we might not blame an agent (corporate or otherwise) for acting irrationally as an accident like we might blame them for acting weakly. So, how can we tell when a corporation is acting weakly, rather than just accidentally irrational? In the case of Chairs 4 All, there is a mismatch perhaps between the judgment by executives of what is to be done (expressed through the mission statement) and the collective conduct of employees. But why think this constitutes an instances of genuine weakness of will, as opposed to a kind of collective irrationality?

Accidents do happen, and we do generally make allowances for this. Chip, as well, may forgive himself for accidentally eating chocolate, if he did so unthinkingly, forgetting his earlier resolution. Still, that accidents happen does not undermine the reality of weak-willed behavior for us or for corporations. If Chip has a habit of ‘forgetting’ his resolution to avoid chocolate at convenient times, then he may well blame himself after the fact for his failure to remain vigilant about his resolution. But there is a hard question about how to distinguish blameworthy weakness from an accident.

There will not a context-free answer to this question. In a given case, we will have to ask: Are these ‘accidents’ common, common enough for it to be a failing not to have done more to guard against it? And how high are the stakes in this case? The higher the stakes, the more we might expect agents to do to avoid accidents. In the case of Chairs 4 All, since the firm’s conduct has not substantively changed in years, we can infer that there is a systemic issue that the firm has failed to address, despite the high stakes.

As a second issue, it will be hard to differentiate a case of corporate weakness from a case where the firm intentionally acted wrongly without ever having sincerely resolved to act rightly. Firms often strike us as disingenuous, and so an unsurprising intuition in the case of Volkswagen or Chairs 4 All is the thought that these firms never *really* intended or resolved to do the right thing. Sure, Chairs 4 All has this nice statement on its website. But if nothing has been done to implement it, then we will doubt whether the firm is sincere in issuing it. If they are not, then their ultimate failure to implement good policies is not merely a failure of weakness; instead, the firm would have intentionally done the wrong thing and be additionally responsible for their disingenuousness.^12^

Again, this is a real concern, and it connects to a larger problem of being able to judge the authenticity of firms. Some have puzzled, for instance, about our demand for public apologies for corporate wrongdoing, yet at our inability to take those apologies as authentic (MacLachlan, 2015). Others have considered whether individuals can ever genuinely *trust* (as opposed to merely rely on) group agents like corporations (Pouryousefi & Tallant, 2023; Tollefsen, 2008). And there is of course a much larger literature within marketing on brand authenticity (e.g., Campagna et al., 2023; Morhart et al., 2015; Södergren, 2021). So, while I cannot resolve this issue here, we can see that there is a lot of work to draw from when thinking about how to differentiate an authentic instance of corporate weakness from disingenuous corporate marketing. Much of this, I suspect, will come down to whether the firm has a history of doing what it says, and whether the supposedly weak-willed behavior is actually in line with what we can infer about the firm’s strategy. If some bad corporate conduct is expedient or easy, and it is a clear departure from the firm’s demonstrated strategy, then it will seem more like a case of potential weakness.

As a connected final issue, we may wonder how to judge when there is an instance of corporate weakness as opposed to a case where the corporation has not intentionally acted at all. Imagine a case where managers make a certain decision, but rogue employees do as they please. This certainly happens, and there are times where employees act quite badly against the firm’s interests. So, what is weak conduct of the firm versus mere bad, non-conforming conduct done by employees, where the firm is not *really* in control, and individuals should be responsible rather than the firm?

Frankly, this is one of the hardest questions for any proponent of CMR to answer. I take it that we are committed to *some* answer; proponents will say that employees can remain responsible for their conduct even as employees, but firms *too* can be responsible, even if they are not responsible for all employee conduct. And, crucially, corporate responsibility is not reducible to the responsibility of employees. So, proponents need to think that there can be some way in principle to disengage the appropriate attributions of responsibility. I do not have one to hand at present, but there are a few things to be said.

It bears acknowledging that this issue does seem pressing in the corporate case in a way that it is not for individuals. Unless we are convinced individuals are themselves best modeled as groups (Dietz, 2020), there is a special challenge with how firms should handle the autonomy of their members, and how they could be responsible for employee conduct (especially in cases where employees seem to be acting against the explicit directives of the firm). With that said, there *is* an analogous problem that we face in the case of individuals.

If I bite my nails when I have resolved not to, then we may take me to be intentionally acting weakly. At some point, though, we may think that I am so inexorably drawn to nail-biting that it should be understood as a *compulsion*. And, traditionally, it has seemed inappropriate to hold people responsible (at least to the same degree) for compulsive behavior. How are we to distinguish weak-willed behavior from mere compulsion? This is a similar question to the one above, and yet it is one of the infamously challenging issues in the literature on weakness of the will.

A standard answer to how to distinguish weakness from compulsion adverts to how *resistible* the behavior in question is. The more resistible it is, and the more under your control it is whether you perform it, the more we will want to say that it is not compulsion, and that you are directly responsible for it. This is what Zaragoza (2006) calls the ‘standard view’ (*c. f.* Gorman, 2023). In the corporate case, this will come down to our judgments about how under the control of the firm the bad conduct was or could have been.

This solution involves again drawing us to think about organizational control,^13^ and it also permits us to recognize that there might be an appropriate amount of organizational control of employees, where absolute control is not necessary. Biting my nails is under my control in the sense that I could strap myself down—as Ulysses to the mast—to avoid it. It is resistible in some absolute sense, but we would typically still classify it as compulsive and not hold ourselves responsible for failing when this level of self-control is needed. Similarly, we will have to determine how much employee conduct can be controlled versus how much of it *should* be controlled.^14^

This is a process that firms should be happy to facilitate. Firms do not want to be blamed for bad conduct, but firms *do* want to be praised for good conduct. But justifiably praising a firm also involves attributing the conduct to the firm itself, rather than its agents. So, while firms will not want to be responsible for *all* employee conduct, they should endorse a standard of organizational control that leaves them sufficiently praiseworthy and blameworthy alike. (Where should the standard be? I cannot fully address this, but I am open to skepticism about there being any particular, universal answer.) When we reflect on this question of the right standard of organizational control, though, it leads us to an interesting issue around how best to address corporate weakness, and so it is to this that we will now turn.

### A Challenge for Correcting Corporate Weakness

A natural thought might be that corporate weakness is a matter of the firm’s being insufficiently or inadequately controlled. However, as we just saw, weakness is not necessarily about being *out of control*. If conduct is truly out of your control, then it is hard to see how it can even be attributed to you as an agent. It is often clear in cases of weakness of will that the agents involved acted in ways that were certainly under their control (Tappolet, 2017). Instead, with these cases there seems to be a failure to be *well*-controlled.

Still, someone may think that what it is to be well-controlled as an organization is to exert *tighter* control over it, to control it to a greater degree, or ensure more centralized control. This may involve having compliance programs built to be measurably effective, as opposed to merely shielding the firm from liability (Chen & Soltes, 2018). To ensure ethical conduct, we may think to compel employees to further undergo specific ethics programs that tell them how to react in certain situations. And the firm may need to engage in even more employee monitoring or conduct policing of employees with thorough rulebooks and procedures. But while firms should strive for efficacy in their compliance and ethics programs, it may be that there are reasons why a firm should not exert this level of control.

Exerting this much control will likely involve invading the privacy of employees or acting tyrannically toward them. It exercises domination. That organizational control has this nefarious potential (or reality) has long been discussed (e.g., Goldman & Van Houten, 1977; Jermier, 1998). Apart from being likely morally wrong to exert this level of control, it’s expensive. It can also make likely other kinds of moral failures. For instance, Stansbury & Barry (2007) discuss how ethics programs that control employees but might lead to worse behavior, potentially making employees less able to handle novel moral situations. So, brute control of employees is both no guarantee of success, and it may separately wrong employees in the process.

To get a handle on how employees might be appropriately governed to avoid corporate weakness, I want to quickly recognize two sources of corporate weakness, and it will be clear that merely exerting more control over employees will not correct them. Instead, we will see how work at the top of organizations, followed by sufficient communication, is a better strategy to avoid weakness.

First, suppose the firm makes a resolution expressed through the mission statement. How will that resolution be seen through? The firm and its employees are operating with a certain momentum, engaging in business as they always have. So, despite some explicit statement, if there are no mechanisms by which to translate that statement into action, then the firm is likely to fail to conform to it. This is a reasonable problem, as we may give in to temptation because our decisions basically aren’t specific enough about how to fulfill them (Schwenkler, forthcoming).

Here, there is a clear strategic deficit to be filled. If the firm issues an ambitious mission statement, which requires a clear departure with the status quo, then we will charge them with inauthenticity if they make no moves toward seeing it through. But even if some changes are made, the firm can be expected to act weakly unless there is a sufficiently rigorous strategy in place, and where this strategy is sufficiently filled out and communicated to employees, leaving no room for ambiguity.

This is consistent with how some have considered combatting weakness in the case of individuals. Snoek et al. (2016) emphasizes the reliance on diachronic strategies over brute willpower in avoiding weakness. Similarly, to guard against corporate weakness, firms should focus on strategic control systems (Goold & Quinn, 1990; Ittner & Larcker, 1997), or strategic implementation more generally (Tawse & Tabesh, 2021). Or, given that the case involves mission statements, we could focus even more directly on the issue of strategies for their successful implementation (Rey and Bastons, 2018). This may involve some degree of control, but it is not necessarily a matter of exerting *more* control over employees.

Second, suppose the firm makes a resolution expressed through the mission statement, and even delivers a strategy to achieve it; however, suppose that what is expressed seems clearly at odds in various ways with what is promoted or privileged by other policies of the firm. As an employee, it may be clear how I should act to deliver upon this resolution, but that may be incompatible with other actions that would deliver on other aims of the firm. This situation can arise if the practical or value perspective of the firm is insufficiently cohesive. Where there is no coherent value perspective, or where resolutions made are at odds with that perspective, we risk corporate weakness. This is a reasonable problem, as weakness of the will could be a matter of having insufficient coherence of one’s practical identity (Jung, 2020).

Here, the prescription is a shoring up of the value perspective of the firm. Proponents of CMR generally have been amenable to talking about the firm as having a kind of agential or practical perspective, sometimes called the ‘rational point of view’ (Hess, 2010; Rovane, 1998). And a firm’s mission statement can clearly be important to or an articulation of this perspective. However, just how key a mission statement is to the firm’s actual practical perspective may depend on the degree to which it is embedded in the firm.

Mission statements can be tied more closely to the firm’s identity by being engaged with more in reports to stakeholders (Leuthesser & Kohli, 1997). They can also be more effective when managers are sufficiently committed to them (Williams et al., 2014). Mission statements may also be a tool used by managers to align the values of employees with the values expressed in the mission statement (Toh et al., 2022). However, we should be careful, as an imposition of values can again introduces the concern that this involves an inappropriately dominating form of control. While this worry is pressing, it is worth acknowledging that value congruence between managers, the firm, and employees does seem better for everybody. This value congruence results in positive behavioral change for employees (Denisi & Smith, 2014), and it is positively related to the ethical behavior of managers (Posner et al., 1985). Instead of imposing corporate values, employees could be selected into the firm on the basis of value alignment. Though, employees could still feel compelled to shift the appearance of their values on the basis of the precarity of employment.

It may be sufficient if firms engage in communication strategies to clarify to employees a coherent value perspective of the firm (Dermol & Širca, 2018). If employees can be not merely told the mission, but brought to understand how the values it conveys are important and committed to by bosses and peers, then this may help to internalize the mission in their actions (Marimon et al., 2016), even if a given employee does not in fact share these values. At least then, employees would be in a position to judge when their conduct is best in line with these values as they act on the firm’s behalf.

## Conclusion

Those who accept or advocate for corporate moral responsibility are already best positioned to recognize a panoply of corporate wrongs. Instead of viewing all of it as a matter of the firm’s failing to recognize morally relevant considerations, or failing to correctly weigh different moral concerns, we can now see the wide range of cases of corporations appearing to instantiate something like weakness of the will. Though it may not always manifest exactly how it does in us—recognized phenomenologically as a *giving in* to temptation—all proponents of CMR should take firms to be capable of forming resolutions yet failing to live up to them.

Recognizing corporate weakness is important not only because it better explains apparent instances of corporate wrongdoing, but because it might warrant a different kind of response. It could warrant a different response from us in society insofar as wrongdoing committed out of weakness is overall less blameworthy than if the action had been done intentionally and with full resolve. Some have suggested blameworthiness comes in degrees (e.g., Coates, 2019; Nelkin, 2016), and so a more complete business ethics would provide a nuanced response to corporate weakness. This could also warrant a different response within firms. By investigating how weakness comes about, we can develop prospective strategies to avoid it, navigating the levers of organizational control while working to maintain the requisite autonomy and dignity of employees.

## Data Availability

This statement is not applicable for this paper, since it’s a theoretical methodology and has no corresponding data.

## Footnotes

1 One recent exception to acknowledge can be found in Collins (2023: 101–2). Collins discusses organizational akrasia in passing as a part of a larger discussion of how we can ascribe character/character traits to organizations. She generally takes it to be possible, though does not argue the point, and she does slightly earlier take on board French’s claim that organizations are less prone to fickleness (98).

2 Much valuable work has been done within management scholarship on mission statements (e.g., Campbell, 1997; Ireland & Hitt, 1992; Pearce & David, 1987), their relation to performance or financial impact (e.g. Bart & Baetz, 1998; Bart, Bontis, & Taggar, 2001; Desmidt, Prinzie, & Decramer, 2011; Sidhu, 2003), and their efficacy (e.g., Braun et al., 2012; Rey & Bastons, 2018). We are concerned with a broader kind of corporate wrongdoing that transcends failures of mission alignment. But we will return below to focus on how some of this scholarship could be leveraged to avoid corporate weakness.

3 Another way to proceed would be to present a psychological situation that would be puzzling (such as judging A to be better than B but being motivated to do B) and then to argue that this situation is instantiated (Davidson, 1980).

4 It *may* be. All of this is consistent with the idea that employees within firms act akratically, and that corporate behavior is at times instantiated by akratic employees. See Patsioti-Tsacpounidis (2023) for an interesting discussion of corporate wrongdoing grounded in the Aristotelian conception of akrasia, which entirely concerns how employees can akratically engage in wrongdoing. I am certainly not ruling this out or assessing how common it is. But more important for us is the possibility (and, indeed, the easy plausibility) of employees doing what they think is right, which nevertheless leads to corporate akrasia.

5 More recently, Flowerman (2024) also considers how (primarily political) groups can instantiate weakness of will, and she similarly criticizes Pettit’s view as too narrow. However, while Flowerman is more sensitive to more sources of weakness (or ‘akratic breaks’), she does not consider group weakness coming about through habitual agency, which I take to be much more prevalent in group and corporate cases. Also, neither Pettit nor Flowerman discuss the particular challenges around the collective mentality required for weakness, and whether they can be instantiated by corporate actors.

6 I am leaving aside any discussion of weakness of will that follows the Socratic understanding mentioned—of being led to change one’s judgement on the basis of a misperception. If this is a way we act weakly, I doubt it is the only way. And so, if these perceptions require abilities firms lack, their inability to act weakly in this way would not stop them from acting weakly in others. That said, I *do* think that firms can act weakly in this way. While we often talk about conscious perceptions, not all perceptual experiences are conscious. And I do think it is possible for firms to be led to misrepresent some opportunities to management in ways that lead to wrongly abandoning resolutions.

7 There is quite a large literature about precisely how to understand the nature of these states within groups and corporations. Given this, there are of course nuanced positions that could raise trouble. For example, we might think that firms are capable of instantiating the attitude of *acceptance*, but not *belief* (Wray, 2001, 2003). If we thought that weakness is a matter of acting against one’s judgment of what is best, and we thought that judgment is a kind of belief, then corporations would technically not be able to act weakly. Here, one could argue against these considerations positively for group belief, or one could consider more closely the nature of judgment. I am inclined towards the former approach, but this need not be seen through here. The more important point is that most proponents of CMR do take firms to have these states, and so such proponents should accept the possibility of corporate weakness.

8 French also maintains that firms can be built to avoid *our* weakness. And, again, perhaps. But two points. First, and more importantly, this is to some extent irrelevant. As we saw in discussing Pettit, proponents of CMR should not think that *corporate* weakness is necessarily a matter of the weakness of employees. Second, though, I again doubt that firms genuinely have been built in this way to avoid our weakness. In some sense, how could they be? Our individual weakness of will is just too personal. Depending on my own view of what I ought to do, it could be that even coming to work and doing my job efficiently *is* my acting weakly. (Perhaps I judge all things considered that I should shirk on the job or quit.) What French likely means is that firms can do a lot to monitor and police the conduct of their employees, ensuring that employees do not go rouge in the way that Pettit discusses. That may be, although avoiding rouge employees may still not avoid the kind of corporate habitual weakness discussed below.

9 While my focus here is on how firms instantiate weakness as a matter of habitual agency, we might be tempted to think that surely the most common form of corporate weakness is of the Aristotelean variety. Firms come to judgments, but then there is insufficient motivation stemming from those judgments to get the firm to cooperate. This may indeed often be the case. On the Aristotelian picture, the agent is motivated by other of their attitudes rather than being motivated by their judgment that a certain action is the right one. And perhaps individuals in the firm are directly sensitive to the pressure on the firm to cutting cost or upping revenue even as the firm has centrally decided to act in ways that will increase costs or suppress revenue. I focus more here on corporate habitual agency because I think corporations are more prone to weakness in this way than we are, and there are interesting interdisciplinary resources that can be brought to bear to characterize this form of corporate agency.

10 While routines may constitute corporate habits, we need to be careful here. First, routines have been spoken of in many ways (Becker, *op. cit.*), as have habits (Makowski, *op. cit.*). Some of these ways lend themselves to this idea better than others. For example, some identify routines with patterns of action within the firm (e.g., Parmigiani & Howard-Grenville, 2011; Petland & Hærem, 2015), but even this may not constitute corporate habit, if not all of the elements of this pattern of employee action constitutes a pattern of conduct of the firm itself.

11 Some opponents of corporate moral responsibility deny it precisely because they think corporations can never adequately assume the necessary type of control (McKenna, 2006: 31), or because employees are not able to switch between acting for their own reasons versus taking on corporate reasons (Leffler, 2024). In contrast, Goodpaster (1979), Donaldson (1982), and Silver (2022), among others argue that corporations can be sensitive to and act for their own moral reasons.

12 Whereas is it all too common to expect this kind of behavior, there has been little work on group or corporate lying in particular. As one recent exception, Lackey (2020) discusses lies and bullshit in the context of group assertions, and in doing so raises a challenge for popular views of group belief. See de Ridder (2022), Marsili (2023), and Hormio (2022) for responses. Her objections do not hold for more directly functionalist or interpretivist views of corporate attitudes (views that maintain that mental states ascriptions are accurate purely on the basis of the firm’s satisfying the right kind of functional characterization), which have often been more popular for proponents of corporate agency (e.g., Collins, 2023; Tollefsen, 2015). My response (below) to how to potentially judge corporate sincerity in some way relates to how Hormio, (2022)(*op. cit.*) discusses the significance of narrative coherence, where for me this coherence is found in coherence with the firm’s strategy.

13 That it connects with this broader topic is why I focus on it here. However, there are challenges for and alternatives to this view, and it is an open question how best to draw this distinction, whether it should be drawn the same across the individual and corporate context, and what the upshots are for going one way rather than another. Zaragoza (*op. cit*.) argues that the difference lies in the fact that compulsion involves the presence of certain phenomenological features (of discomfort). If that is true, that firms will never count as acting compulsively if they lack phenomenology. Though, they may still act weakly, and this may still be differentiated from a situation with rogue employees.

14 Perhaps all employee conduct done beyond the scope of corporate control counts as corporate compulsion. It will not be intentional behavior of the firm, and it will not count as corporate weakness. But this is not to say that the firm cannot still be responsible for it. If the firm has failed to exercise a duty of collective self-control, or self-induced its own moral incapacity (de Haan, 2023), it may seem *vicariously* responsible for the conduct of its agents. It is unclear exactly what we *get* from the recognition of corporate vicarious responsibility. It is commonly used within the criminal law to hold firms legally responsible, but there is reason to be skeptical that it should play this role (Silver, forthcoming). However, we could still take vicarious responsibility as moral responsibility of some kind (Goetze, 2021; Mellor, 2021), and so this could still play an important social function. (Alternatively, we may hold firms directly responsible for creating the conditions for the bad behavior [Blomberg, 2023]).
